# Identification of Key Genes Related With Aspartic Acid Metabolism and Corresponding Protein Expression in Human Colon Cancer With Postoperative Prognosis and the Underlying Molecular Pathways Prediction

**DOI:** 10.3389/fcell.2022.812271

**Published:** 2022-01-31

**Authors:** Weixuan Sun, Chaoran Jia, Xiaojun Zhang, Zhaoyi Wang, Yaping Li, Xuedong Fang

**Affiliations:** ^1^ China-Japan Union Hospital of Jilin University, Changchun, China; ^2^ Northeast Normal University, Changchun, China; ^3^ The Second Hospital of Jilin University, Changchun, China

**Keywords:** consensus clustering, colon cancer, immune infiltration, prognostic signature, aspartic acid metabolism

## Abstract

**Objective:** Colon cancer is one of the most frequent and lethal neoplasias. Altered metabolic activity is a well-known hallmark for cancer. The present study is aiming to screen key genes associated with tumor metabolism and construct a prognostic signature of colon cancer patients.

**Methods:** Glutamine- and UC- metabolism related genes were downloaded from GSEA MsigDB. Three key genes were screened by Cox regression analysis with data samples downloaded from TCGA and GSE29623 database. Consistent clustering based on the prognostic genes identified was employed to divide the colon cancer samples into two clusters with significant OS differences. The mRNA and protein expression of the key genes in colon tissues and matched adjacent noncancerous tissues of 16 patients were detected by IHC, qPCR, and Western blot to validate the constructed clustering model. GO, GSVA, and IPA were used to predict the relevant metabolic pathways.

**Results:** According to the three key genes identified, i.e., ASNS, CEBPA, and CAD, the cohort can be divided into two clusters with prognosis differences. Clinical specimen results confirmed that the risk model established was effective, and the different expression pattern of ASNS and CEBPA was correlated with TNM stage and lymph node metastasis, whilst that of CAD was correlated with post-operative tumor metastasis and recurrence. Molecular mechanism prediction indicated that CREB, insulin, and RNA Pol II were the key nodes affecting CEBPA and ASNS expression. Moreover, TIDE algorithm reflected the better immune response of the cluster with shorter OS. Further immune infiltration and checkpoints analyses provided important reference for clinicians to perform individualized immunotherapy.

**Conclusion:** Differential expression profile of three aspartic acid metabolic-associated genes, ASNS, CEBPA, and CAD, can be considered as a risk model with a good evaluation effect on the prognosis of colon cancer patients.

## Introduction

After dismissing the revelation of the “Warburg effect” for almost a century, the study of metabolic reprogramming has been revived as the understanding of cancer occurrence, development, and metastasis mechanism deepened (Ward and Thompson, 2012). Aside from the fact that tumor cells rely on aerobic glycolysis rather than the mitochondrial oxidative phosphorylation to survive (Vander et al., 2009), other metabolic alterations on malignant tumors have been gradually discovered, including both the lipid and amino acid metabolisms. Lipid uptake and storage are elevated in malignant tumors, while lipogenesis is strongly up-regulated to accommodate increased membrane biogenesis (Röhrig and Schulze, 2016; Pascual et al., 2017). Regarding the reprogramming of amino acid metabolism, glutamine, the most abundant circulating amino acid, is one of the hubs linking the metabolic processes (Altman et al., 2016). The high glutamine level in the blood provides carbon and nitrogen for cells. The glutamine-derived carbon can be used to synthesize amino acids and fatty acids, while the nitrogen from glutamine directly affects the biosynthesis of purines and pyrimidines (Rubin, 1990; Lane and Fan, 2015). Abnormal changes in glutamine related metabolic processes are considered to be hallmarks of tumorigenesis. Under glutamine deficiency condition, asparagine can complement glutamine as an important nitrogen source to support tumor cell proliferation (Zhang et al., 2014). Since glutamine is often found to be limited in the tumor microenvironment (TME), asparagine can even support epithelial breast cancer cells to proliferate in the absence of exogenous glutamine. In this context, asparagine supports *de novo* biosynthesis of glutamine through enhancing the expression of glutamine syntheses (Pavlova et al., 2018). And asparagine restriction reduces the expression of genes involved in the epithelial-mesenchymal transition, a key step for metastasis to initiate (Knott et al., 2018). While glutamine metabolism is crucial for ammonium assimilation, UC is the main pathway converting excess nitrogen into disposable form, which is an important process for ammonium alienation. UC dysregulation is considered to be a general tumor metabolic hallmark based on recent discoveries, for instance, UC rewiring toward pyrimidine synthesis promotes carcinogenesis (Krebs, 1970; Spinelli et al., 2017).

It is noteworthy to mention that other than tumor cells, the immune cells located in the surrounding TME also undergo amino acid metabolic reprogramming. For example, glutamine levels alteration can activate the tumor-infiltrating T cells and influence tumor growth by directly contacting or stimulating other cells in the TME (Keshet et al., 2018).

As one of the most prevalent and high-incidence cancers, CRC is a very heterogeneous disease that develops through a gradual accumulation of genetic and epigenetic changes (Simon, 2016). Although inexhaustive, some initial attempts have been made to uncover the mechanism of metabolic reprogramming in CRC (Renner et al., 2017). Typically, metabolomics analysis of serum/plasma samples from patients revealed the six most affected metabolic pathways in CRC, including protein biosynthesis, glutamine metabolism, ammonia cycle, alanine metabolism, aspartic acid metabolism and citric acid cycle (Wilkinson et al., 2010).

Indeed, CRC comprises both colon cancer and rectal cancer because they share many physiological features. Compared with that of rectal cancer, the early diagnosis and treatment of colon cancer patients shows much better prognosis (Freeman, 2013). However, in many patients with colon cancer, TNM staging-the existing “gold standard” prediction model-does not accurately predict tumor recurrence, metastasis, or survival. TNM staging is suboptimal as seen by the variation in target-therapy outcomes that exists amongst patients of the same stage. Thus, we employed bioinformatics techniques to obtain critical nitrogen metabolism-related genes and explored their survival predictive potential by classifying the colon cancer patients into two clusters. We further compared the underlying molecular mechanism and the immune behavior of the two clusters, and indicated their benefits for individualized target- and immuno-therapy.

## Materials and Methods

### Data Collection

The transcripts and complete clinical data of 415 patients were downloaded from the Cancer Genome Atlas (TCGA) database. Gene Expression Omnibus (GEO) data source: GSE29623, 67 cases of colon cancer samples with complete clinical data were selected to verify the results of TCGA data analysis. The gene sets of UC metabolism and glutamine metabolism were downloaded from GSEA MsigDB. Gene set of UC metabolism: GO_ UREA_ CYCLE, GO_ UREA_ METABOLIC_ Process, including 13 genes. Glutamine metabolism gene set: GO_ GLUTAMINE_ METABOLIC_ Process, including 23 genes.

### Cox Regression Analysis

Univariate Cox regression analysis was employed to select the genes with *p*-value < 0.2 by the proportional hazards model. The selected genes were incorporated into the multivariate Cox regression analysis, and genes with *p*-value < 0.05 were selected as prognostic genes. Cox regression analysis was used to determine the independent clinical risk factors, including patient age, gender, tumor TNM staging, etc.

### Consistent Clustering

Consistent clustering of the TCGA and GEO databases were based on the prognostic genes obtained previously. The software package “ConsensusClusterPlus” was used for consistent clustering, and the optimal number of subgroups was evaluated using cumulative distribution function and consensus matrices (Li et al., 2014). Moreover, t-distributed Stochastic Neighbor Embedding (t-SNE) clustering algorithm was conducted to verify the clustering results.

### Gene Ontology, Gene Set Variation Analysis, and Ingenuity Pathway Analysis Enrichment Analysis

Using the limma package, the differential expression of 415 different TCGA samples was analyzed, and the cutoff criteria set as log2FoldChange <0.5 and false discovery rate (FDR) < 0.05. The GO system comprised three sections: biological process, molecular functions, and cellular components. To further explore the functions of the differential genes, GO function enrichment analysis was performed using the cluster profiler software. The screening condition was |t-value| > 2 and *p*-value < 0.05 (Hänzelmann et al., 2013). The GSVA is a non-parametric unsupervised analysis method, mainly used to evaluate the gene set enrichment of chips and transcriptomes. GSVA transforms a matrix of “sample × gene” into a matrix of “sample × pathway”, which directly reflects the connection between the sample and the reaction pathway. Similarly, using the limma package to do the same analysis on the results of GSVA (still a matrix) can find pathways with significant differences between samples. A more comprehensive functional enrichment analysis was conducted using the IPA software to elucidate the affected genes and signal pathways.

### XCell Immune Infiltration Analysis

XCell (http://XCell.ucsf.edu/) is a single sample Gene Set Enrichment Analysis (ssGSEA) based approach. The abundance scores of 64 immune cell types were enriched by XCell (Aran et al., 2017). TIDE (tumor immune dysfunction and exclusion) (http://tide.dfci.harvard.edu) algorithm was used to predict the immunotherapy response of each patient.The statistical model of TIDE was trained on clinical tumor profiles without immune checkpoint blockade treatments since the immune evasion mechanisms in treatment-naïve tumors are influence patient response to immunotherapies (Jiang et al., 2018).

### Patients Specimens and Clinical Data Collection

The primary colon cancer tissues and corresponding adjacent nontumor samples were retrieved from the China-Japan Union Hospital of Jilin University between January 2016 and January 2017. The eligibility criteria for the specimens are as follows: 1) patients aged 55–70 who received radical colon cancer surgery without any chemotherapy, radiotherapy or other intervention within 3 years; 2) no history of serious basal metabolic disease; 3) patients with follow-up information for more than 3 years available to determine whether other organ metastasis or recurrence occurred. A total of 16 colon cancer patients meeting the above conditions were screened. Specimens from these patients satisfied these criteria were immediately stockpiled at −80 after surgery and progressed for analysis. All patients were confirmed as colon cancer by postoperative histopathological examination, and the type identification and TNM staging were carried out by the pathology department of the China-Japan Union Hospital of Jilin University. Besides, the clinical data of patients were acquired from the electronic medical record of the department of gastrointestinal colorectal and anal surgery of China-Japan Union Hospital of Jilin University.

### Immunohistochemistry

The slides were incubated at 65°C for 45 min and then dewaxed. Subsequently, we retrieved the antigen using citrate buffer and blocking endogenous peroxidase with 3% H_2_O_2_. After nonspecific antigen blocking, the slides were incubated with antibodies at 4°C overnight. The antibodies against ASNS (dilution 1:200) and CEBPA (dilution 1:200) were purchased from Cellsignal (92479S; 8178S), and the antibody against CAD (dilution 1:200) was purchased from Proteintech (16617-1-AP). The next day, slides were incubated with secondary antibody labeled with Horseradish Peroxidase (HRP) at room temperature for 1 h. Finally, the reaction was visualized using Diaminobezidin and the slides were counter stained with hematoxylin. The digital scanner of tissue section was used to collect the image on the immunohistochemical section. The Seville image analysis system was used to automatically read the tissue measurement area, as well as the number of weak, medium, and strong positive cells in the measurement area (negative without coloring, 0 point; weak positive light yellow, 1 point; medium positive brown yellow, 2 points; strong positive brown, 3 points). The total number of cells were analyzed and calculated respectively. The positive cell rate reflecting the protein expression on the section was also calculated.

### qRT-PCR

Primers used for qPCR were designed using the Primer 5.0 gene primer design software. All primers were synthesized by Comate Bioscience Company Limited (Jilin, China) (ASNS-F: CTT​TTA​TCA​GGG​GGC​TTG​GAC, ASNS-R: CAG​TAA​ATC​GGG​GCT​GTC​TTC; CEBPA-F: CAC​ACC​AGA​AAG​CTA​GGT​CGT​G, CEBPA-R: AAT​GCT​GAA​GGC​ATA​CAG​TAC​AAA​C; CAD-F: CCA​GAC​GTT​GCT​GAT​CAA​CCC, CAD-R: CCA​CAC​CAC​AGT​TCA​GAG​CAG​TC). The mRNA expression levels were detected in triplicate using the SYBR Green Ⅰ dye method. The β-actin was used as a reference gene. The RT-PCR reaction mixture consisted of cDNA (1 μl), PCR-Master Mix (5 μl), PCR-F-Primer (0.5 μl), PCR-R-Primer (0.5 μl), and RNase-free H_2_O (3 µl) in a total volume of 10 μl. The RT-PCR reaction conditions were as follows: denaturation at 95°C for 1 min, followed by 40 cycles of denaturation at 95°C for 15 s, annealing at 60°C for 15 s, and extension at 72°C for 15 s. At the end of the reaction, the melting curve was recorded for the 60–95°C temperature range, and the reaction products were stored at 4°C. Data from the Eppendorf real-time PCR instrument (Hamburg, Germany). We obtained the mRNA CT value of the target gene through qPCR, and then calculated it with the mRNA CT value of the GAPDH, and finally obtained the relative expression of the mRNA of the target gene. The specific formula is as follows:

△CT = CT target gene -Ct GAPDH; △△CT = △CT experimental-△CT control; Relative expression = 2^-△△CT

### Western Blotting

Total protein from tissue samples or cells was extracted and protein concentrations were quantified using a BCA assay kit (Beyotime Biotechnology, China). Then protein samples were electrophoresed on 12% sodium dodecyl sulfate-polyacrylamide (SDS-PAGE) gels and subsequently transferred to polyvinylidene difluoride (PVDF) membranes, which were blocked with 5% skimmed milk and incubated at 4°C overnight with targeted primary antibodies on a rotating wheel. The membranes were washed three times and immediately following the incubation with corresponding HRP-conjugated secondary antibodies for 1 h and then imaging. The gray value was calculated by the AlphaEase FC software. The ratio of the gray value of the target band to the corresponding internal reference β-Actin reflected the relative expressions.

The antibodies against ASNS (dilution 1:500) and CEBPA (dilution 1:500) was purchased from Cellsignal (92479S; 8178S), the antibody against the CAD (dilution 1:500) was purchased from Proteintech (16617-1-AP), and the antibody against β-Actin (dilution 1:500) was purchased from Servicebio (GB12001).

## Results

### Construction of a Cluster Model Using Three Key Genes Identified, i.e., ASNS, CEBPA, and CAD, for Colon Cancer.

**FIGURE 1:**
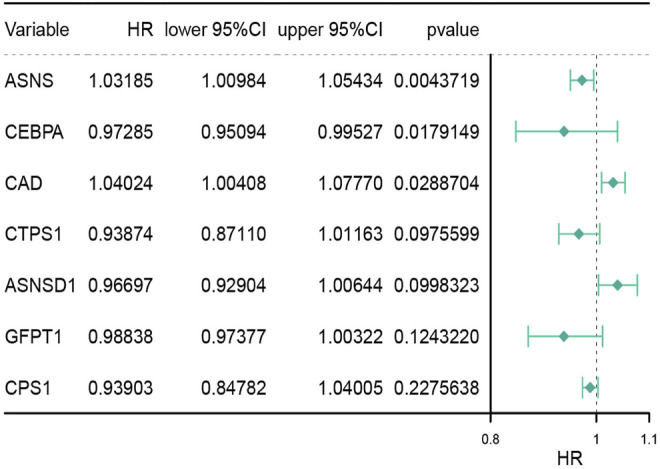
Multivariate analysis of genes associated with glutamine metabolism and the urea cycle to identify key genes. HR stands for risk ratio, the lower/Upper 95% CI was the 95% confidence interval of the risk value. *n* = 415.

**FIGURE 2:**
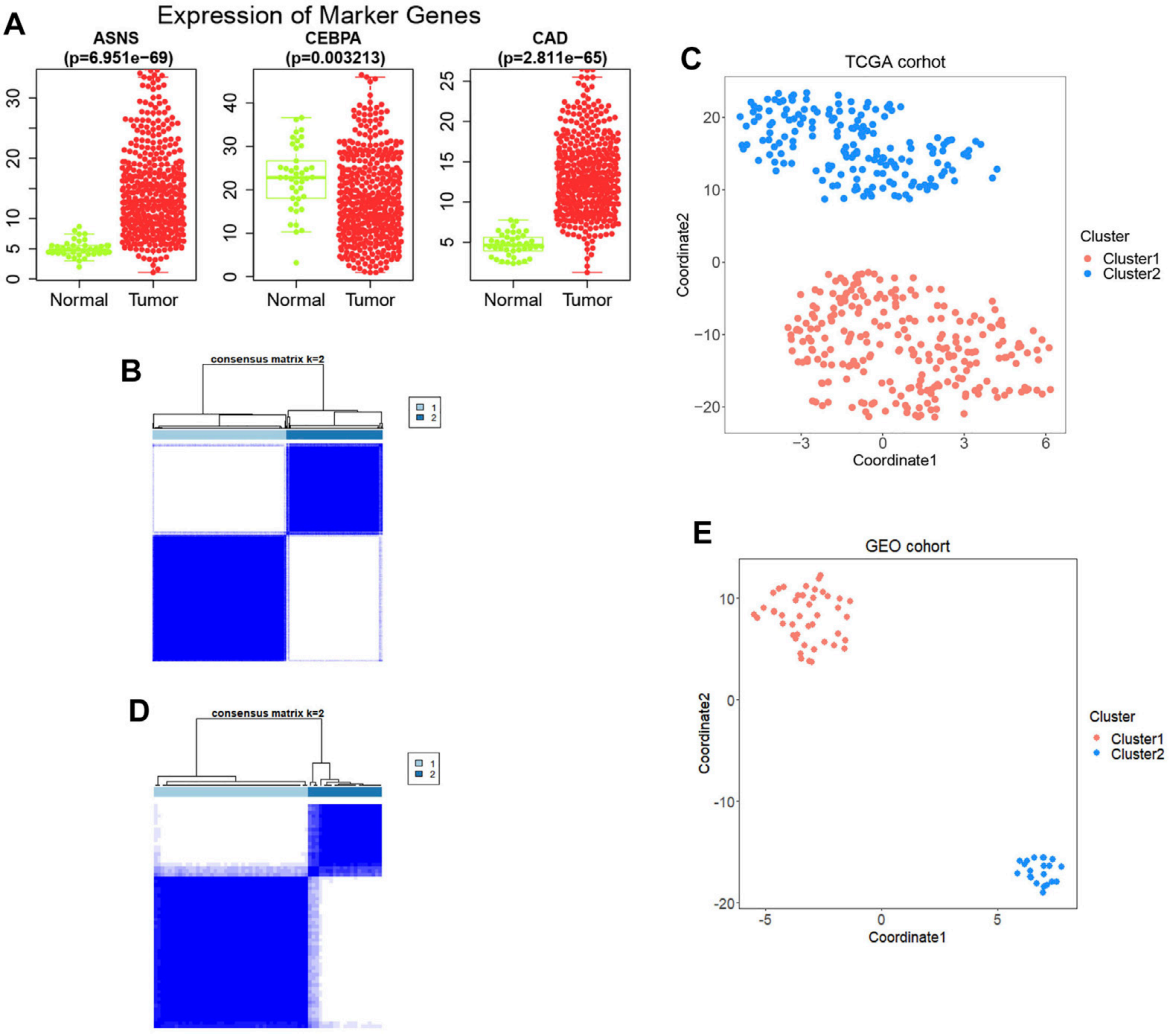
**(A)** The expression levels of the three genes obtained between tumor samples and normal control samples. **(B)** Construction of the prognostic signature based on TCGA cohort and consensus clustering. The cohort was divided into two distinct clusters when *k* = 2. **(C)** The tSNE clustering algorithm divides colon cancer samples from TCGA into two clusters. **(D)** Construction of the prognostic signature based on GSE29623 cohort and consensus clustering. The cohort was divided into two distinct clusters when *k* = 2. **(E)** The tSNE clustering algorithm divides colon cancer samples from GSE29623 into two clusters.

Thirty-six genes related to glutamine metabolism and UC metabolism were obtained from GSEA MsigDB database. These genes were analyzed by univariate Cox regression analysis in 415 colon cancer samples from TCGA database. 11 genes with *p* < 0.2 related to OS were screened (Supplementary Figure S1). Then the 11 genes were analyzed by multivariate Cox regression analysis. Three genes with *p* value <0.05 related to OS were screened (Figure 1; Supplementary Figure S2), i.e., ASNS, CEBPA, and CAD, as the key genes to differentiate the cohort. Their expression was significantly different in colon cancer and normal colon samples in TCGA (Figure 2A).

Based on the expression profiles of ASNS, CEBPA, and CAD, 415 TCGA colon cancer samples were analyzed by consistent clustering (Figure 2B) and tSNE algorithm (Figure 2C), and the samples were divided into two clusters, Cluster 1 and Cluster 2, with longer and shorter survival time, respectively. In order to verify our clustering results, we applied the three genes obtained for consistent clustering and tSNE algorithm analysis in 67 colon cancer samples from GSE29623, and stratified the cohort into two clusters successfully (Figures 2D,E). However, the specific expression of ASNS, CEBPA, and CAD in the two clusters remains unclear.

### Comparision of OS Differences and Clinical Risk Scores Between Each Two Clusters Obtained From TCGA and GSE29623 Databases.

**FIGURE 3:**
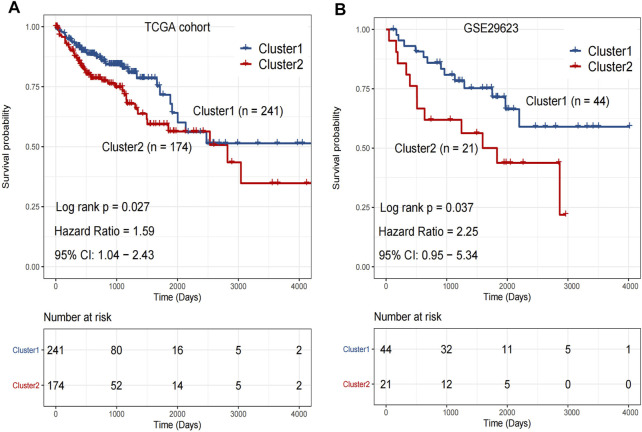
**(A)** The overall survival rate of Cluster 1 was significantly longer than that of Cluster 2 in TCGA. *p* < 0.05. **(B)** The overall survival rate of Cluster 1 was significantly longer than that of Cluster 2 in GSE29623. *p* < 0.05. Analyzed using unpaired *t*-test.

Kaplan-Meier analysis was performed on the clusters of colon cancer samples from TCGA (Cluster 1: n = 241 v.s. Cluster 2: *n* = 174) and GSE29623 databases (Cluster 1: *n* = 44 v.s. Cluster 2: *n* = 21). The OS of Cluster 2 was significantly lower than that of Cluster 1 in both databases (Figures 3A,B).

The clinical risk features for each colon cancer patient included age, gender, tumor grade, stage, etc. The colon cancer samples in TCGA were analyzed by Cox regression. Univariate cox regression analysis showed that tumor stage, T stage, N stage, M stage, and our clustering were risk factors associated with clinical prognosis (Supplementary Figure S3).

### Validation of the Constructed Cluster Models With Clinical Samples.

We further examined the expression of ASNS, CEBPA, and CAD in tumor tissues and adjacent normal tissues from different colon cancer patients. It was verified that our clustering model could divide colon cancer patients into two clusters with different clinical characteristics.

**FIGURE 4:**
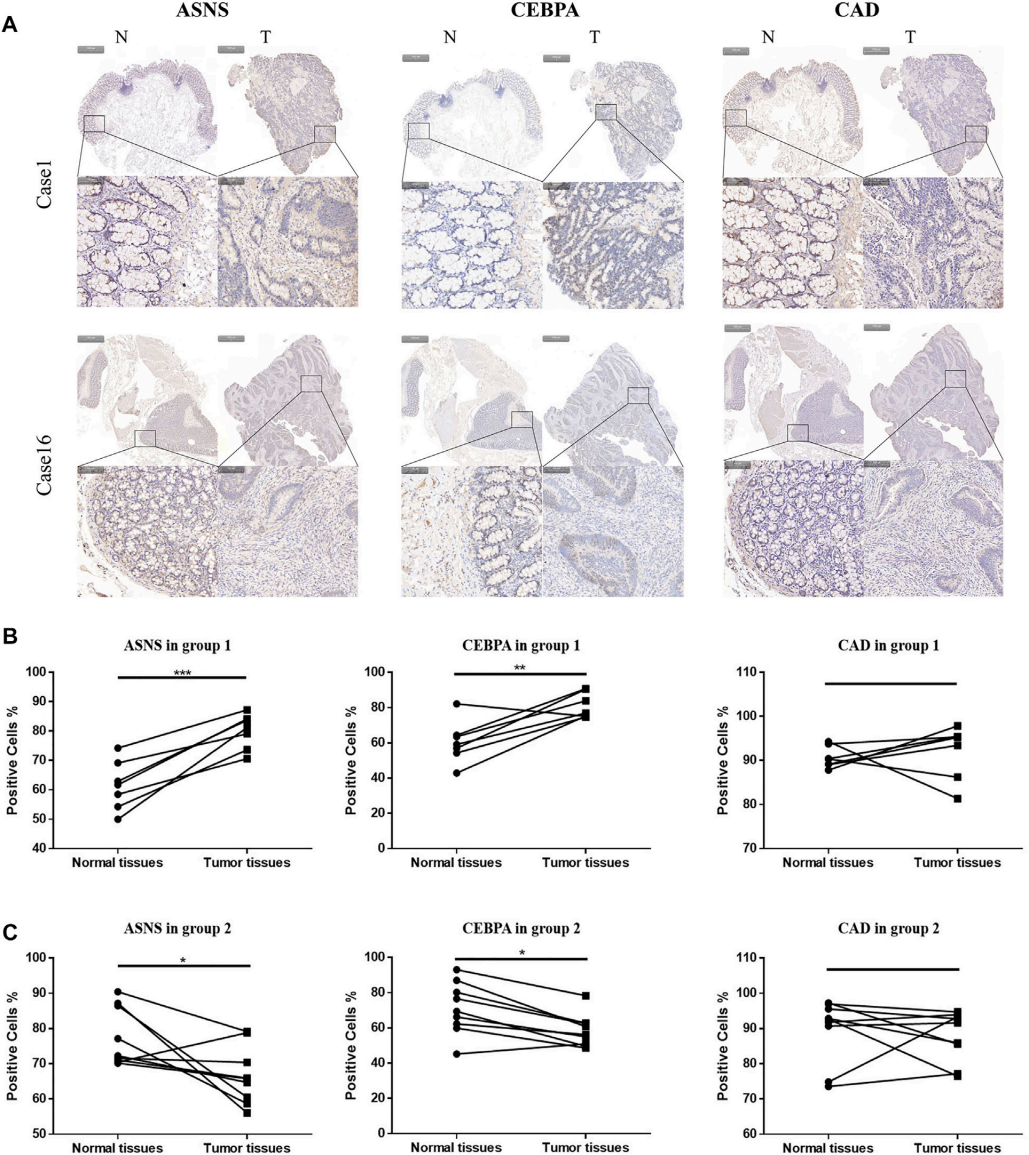
**(A)**Typical images of IHC in 16 pairs of colon cancer tissues showing the protein expression of ASNS, CEBPA and CAD in colon cancer tissues and adjacent nontumor tissues. Up panel, scale bar 1000 µm; down panel, scale bar 100 µm. **(B)** Immunohistochemical positive cell rates of ASNs, CEBPA, and CAD in 7 colon cancer tissues and adjacent nontumor tissues in group 1. **(C)** Immunohistochemical positive cell rates of ASNS, CEBPA and CAD in 9 colon cancer tissues and adjacent nontumor tissues in group 2. *p < 0.05, **p < 0.01 and ***p < 0.001, analyzed using unpaired *t*-test. Group 1, n = 7; Group 2, *n* = 9.

More specifically, 16 pairs of primary colon cancer tissues from the Department of Gastrointestinal Colorectal Surgery in China-Japan Union Hospital of Jilin University were analyzed by IHC staining (Figure 4A). Among them, patients 1–7 (Group 1) had no lymph node metastasis (N0 stage), and patients 8–16 (Group 2) had lymph node metastasis (N1 or N2 stage) (Table 1). Results showed that in Group 1, the protein level of ASNS and CEBPA was higher in tumor tissues than in adjacent normal colon tissues, while in Group 2, the results were opposite. As for the CAD expression, the level in the tumor and non-tumor normal tissues was similiar in both Group 1 and Group 2 (Figures 4B,C).

**TABLE 1:** Clinical information on selected colon cancer patients for validation, which were divided into two groups, i.e., Group 1 and Group 2, according to different N stages.

Sample ID	Case 1	Case 2	Case 3	Case 4	Case 5	Case 6	Case 7
Gender	M	M	M	F	M	M	M
Age	69	65	64	67	63	61	59
Tumor size (cm)	20	12	24	31.5	5.25	11.76	2.4
TNM stage	T3N0Mx	T3N0Mx	T3N0Mx	T3N0Mx	T3N0Mx	T3N0Mx	T3N0Mx
Date of surgery	2016.5	2016.5	2016.6	2016.7	2016.9	2016.11	2017.1
Metastasis or recurrence within 3 years	Occurrence	Not occurrence	Occurrence	Not occurrence	Occurrence	Not occurrence	Occurrence
Group 1							

Sample ID	Case 8	Case 9	Case 10	Case 11	Case 12	Case 13	Case 14	Case 15	Case 16
Gender	M	F	F	M	F	F	F	M	M
Age	69	69	68	59	67	66	61	69	67
Tumor size (cm)	9	13.13	21	14	6.75	202.5	24	40.5	7.5
TNM stage	T3N1Mx	T3N1Mx	T3N1Mx	T3N2Mx	T4N1Mx	T3N1Mx	T3N1Mx	T3N1Mx	T3N1Mx
Date of surgery	2016.4	2016.5	2016.5	2016.7	2016.7	2016.8	2016.9	2016.11	2017.1
Metastasis or recurrence within 3 years	Not occurrence	Not occurrence	Occurrence	Not occurrence	Occurrence	Occurrence	Not occurrence	Occurrence	Occurrence
Group 2									

**FIGURE 5:**
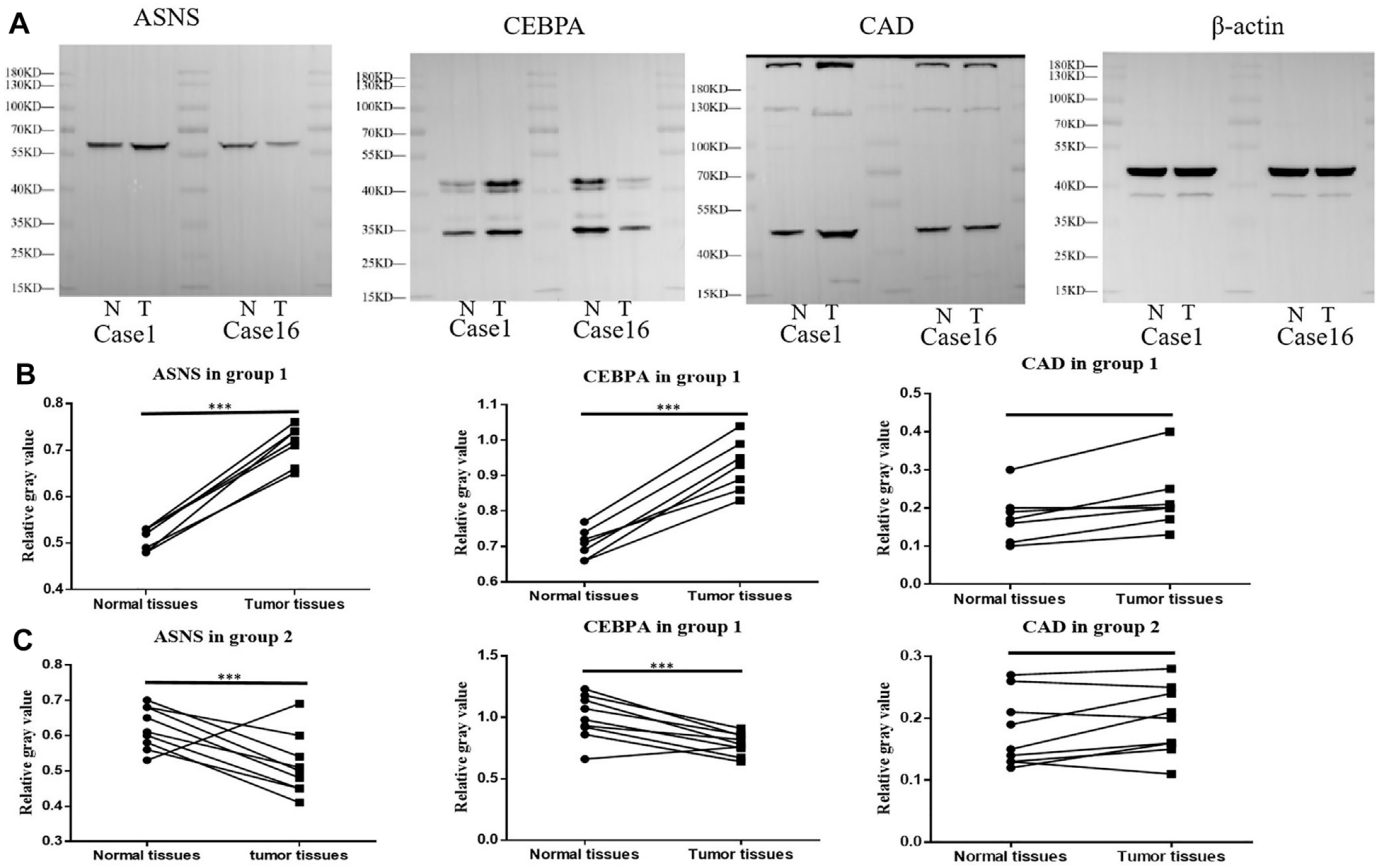
**(A)** ASNS, CEBPA, and CAD expression levels in Case 1 and Case 16 of tumor specimens and adjacent normal specimens. **(B)** The relative gray values of western blot, which represent the relative protein expression level, of ASNS, CEBPA, and CAD in 7 Colon cancer tissues and adjacent nontumor tissues in **(B)** Group 1 and **(C)** Group 2.*p < 0.05, **p < 0.01 and ***p < 0.001, analyzed using unpaired *t*-test. Group 1, *n* = 7; Group 2, *n* = 9.

Further Western blotting and blot gray value analysis were performed to verify the protein expression in 16 pairs of samples (Figure 5A; Supplementary Figure S4). The results were consistent with IHC staining. The expression of ASNS and CEBPA in tumor tissues of Group 1 was higher than that in non-tumor normal tissues, while the expression of ASNS and CEBPA in tumor tissues of Group 2 was lower than that in non-tumor normal tissues. There was no significant difference in the expression of CAD in tumor tissues and non-tumor tissues in different groups (Figures 5B,C). The low expression of ASNS and CEBPA in colon cancer tissues was closely related to the occurrence of lymph node metastasis, which had an impact on prognosis and shortened OS (Freeman., 2013).

The mRNA expressions of ASNS,CEBPA, and CAD in 16 pairs of specimens were detected by qPCR (Table 2), and the correlation analysis was conducted with the clinical characteristics and prognosis of patients. The results showed that the expression of ASNS was correlated with the size of tumor growth,that of CEBPA was correlated with the gender of patients and TNM stage of tumor, and that of CAD was highly correlated with recurrence and metastasis (Table 3).

**TABLE 2:** mRNA relative expression of ASNS, CEBPA, and CAD in Group 1 and Group 2.

Group 1	ASNS		CEBPA		CAD	
	N	T	N	T	N	T
Case 1	1.00	1.67	1.00	1.85	1.00	1.86
Case 2	0.69	1.61	1.52	2.24	0.66	1.06
Case 3	0.97	1.45	0.94	1.72	1.51	0.88
Case 4	0.86	1.98	0.60	2.30	1.78	0.42
Case 5	1.62	1.84	1.77	2.14	1.34	1.55
Case 6	0.66	1.65	0.79	1.92	1.64	0.46
Case 7	1.49	1.63	3.41	2.46	1.07	1.56

Group 2	ASNS		CEBPA		CAD	
	N	T	N	T	N	T
Case 8	1.81	1.54	2.42	1.43	1.17	1.61
Case 9	1.53	1.57	2.04	1.38	1.06	1.61
Case 10	2.07	0.78	2.99	0.59	0.35	2.42
Case 11	1.93	1.62	2.44	1.99	1.65	1.19
Case 12	1.76	0.62	2.17	0.81	1.12	0.58
Case 13	2.19	0.96	2.33	0.84	2.06	0.56
Case 14	1.70	1.41	2.54	2.17	0.92	2.52
Case 15	1.30	1.17	2.33	1.13	1.65	0.73
Case 16	1.81	1.36	2.10	1.31	0.95	1.01

**TABLE 3:** Relationship between ASNS, CEBPA, and CAD expression level and clinicopathological variables in 16 colon cancer patients.

Pathological characteristics	n	Low expression (n)	High expression (n)	*p* value
Age(years)				0.547
≥65	10	3	7	
<65	6	4	2	
Gender				0.174
M	10	3	7	
F	6	4	2	
Tumor size (cm)				0.043
≥20	7	5	2	
<20	9	2	7	
TNM stage				0.674
T3N0Mx	7	3	4	
T3N1Mx	7	3	4	
T3N2Mx	1	0	1	
T4N1Mx	1	1	0	
Metastasis or recurrence within 3 years				0.953
Occurrence	9	4	5	
Not occurrence	7	3	4	

Pathological characteristics	n	Low expression (n)	High expression (n)	*p* value
Age(years)				0.156
≥65	10	3	7	
<65	6	0	6	
Gender				0.01
M	10	0	10	
F	6	3	3	
Tumor size (cm)				0.409
≥20	7	2	5	
<20	9	1	8	
TNM stage				0.034
T3N0Mx	7	0	7	
T3N1Mx	7	2	5	
T3N2Mx	1	0	1	
T4N1Mx	1	1	0	
Metastasis or recurrence within 3 years				0.102
Occurrence	9	3	6	
Not occurrence	7	0	7	

Pathological characteristics	n	Low expression (n)	High expression (n)	*p* value
Age(years)				0.547
≥65	10	5	5	
<65	6	2	4	
Gender				0.174
M	10	3	7	
F	6	4	2	
Tumor size (cm)				0.053
≥20	7	5	2	
<20	9	2	7	
TNM stage				0.674
T3N0Mx	7	3	4	
T3N1Mx	7	3	4	
T3N2Mx	1	0	1	
T4N1Mx	1	1	0	
Metastasis or recurrence within 3 years				0.001
Occurrence	9	1	8	
Not occurrence	7	6	1	

The above experiments have preliminary verified the clustering model. According to the different expressions of ASNS, CEBPA, and CAD, colon cancer patients could be divided into two clusters with different postoperative outcomes.

### Different Expressions of Pathways Related to Biological Function and Substance Metabolism in the Two Clusters

**FIGURE 6:**
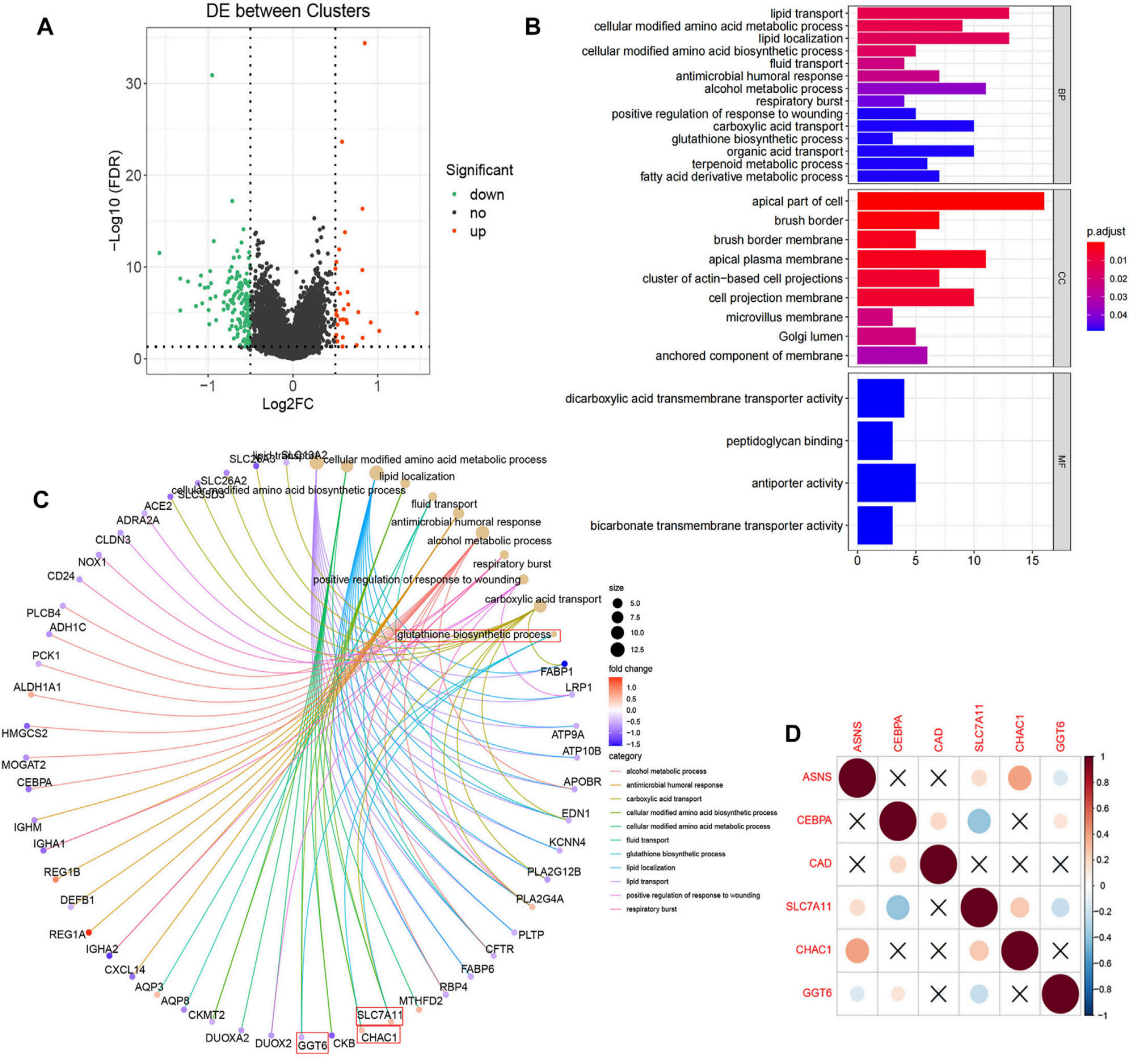
**(A)** Volcano plot displayed the 157 differentially expressed genes of Cluster 2 screened relative to Cluster 1 with 26 up-regulated (red dots) and 131 down-regulated (green dots).The black dots indicate genes that are not significantly different. **(B)** The enriched GO BP, CC, and MF terms of the differential genes. **(C)** The GO entry network diagram, showing the top 11 network relationships of GO enrichment. The process of glutathione synthesis and the genes it contains are indicated with red boxes. **(D)** Correlation diagram of the prognostic genes and glutathione synthesis process enriched genes.

In TCGA database, we describe differences in gene expression between two clusters. Differential expression analysis demonstrated 157 significantly differentially expressed genes, of Cluster 2 relative to Cluster 1, including 26 up-regulated genes and 131 down-regulated genes (Figure 6A). Then GO annotation was performed to understand the influence of these different genes on various functions in the cell. GO BP analysis revealed that these genes were markedly enriched in lipid transport, lipid localization, and alcohol metabolic process. For GO CC analysis, the top three significantly enriched terms were apical part of cell, apical plasma membrane, and cell projection membrane (Figure 6B). We associated the first 11 cell function items obtained by GO analysis with the enriched differential genes (Figure 6C). It is worth mentioning that we enriched the biological process of reduced glutathione. Since reduced glutathione is closely related to glutamine metabolism (glutamine is the precursor of reduced glutathione), we further analyzed the differential genes in the biological process of reduced glutathione, SLC7A11, CHAC1, and GGT6. Correlations between them and the three key genes previously screened were determined (Figure 6D). The results showed that ASNS was positively correlated with CHAC1, while CEBPA was negatively correlated with SLC7A11. This provided the explaination of the correlation between ASNS, CEBPA, and glutathione metabolism.

**FIGURE 7:**
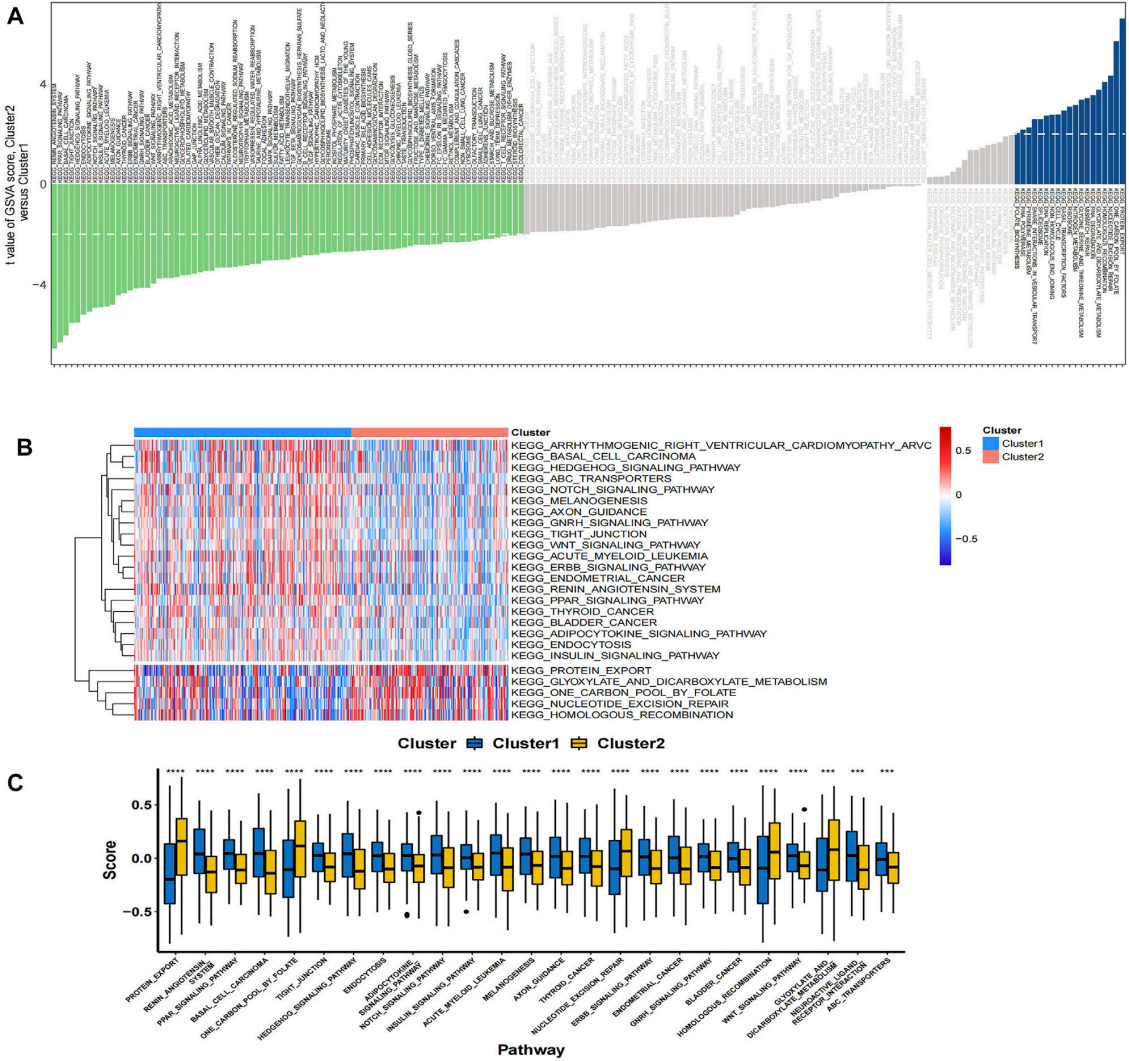
**(A)** The bar plots show the results of GSVA in the TCGA cohort. A negative t-value means that the pathway is enriched in Cluster 1, and a positive t-value means that it is enriched in Cluster 2. **(B)** The cluster heat map of the top 25 enriched KEGG pathways. **(C)** The boxplot showed different activation levels of GSVA-enriched KEGG pathways in Clusters 1 and 2. ^***^ *p* < 0.001, and ^****^ *p* < 0.0001. Analyzed using unpaired *t*-test.

In the TCGA colon cancer sample, GSVA analysis was performed to correlate the differential gene set with the KEGG pathway. It was found that 81 pathways were active in Cluster 1 (T < 2), such as metabolic pathway, cellular signal transduction pathway, and tumor pathway, while 19 pathways were active in Cluster 2 (T > 2), such as glycine, nitrogen and tetrahydrofolate metabolism pathway (Figure 7A). Among the 25 pathways with the largest absolute t value, 20 pathways were activated in Cluster 1 samples, including renin-angiotensin system, PPAR signaling pathway, WNT signaling pathway, etc.; while five pathways were activated in Cluster 2 samples, including protein export, folate carbon pool, nuclear maneuver repair, etc. (Figures 7B,C).

### IPA Gene Interaction Network Prediction of Key Genes ASNS and CEBPA

**FIGURE 8:**
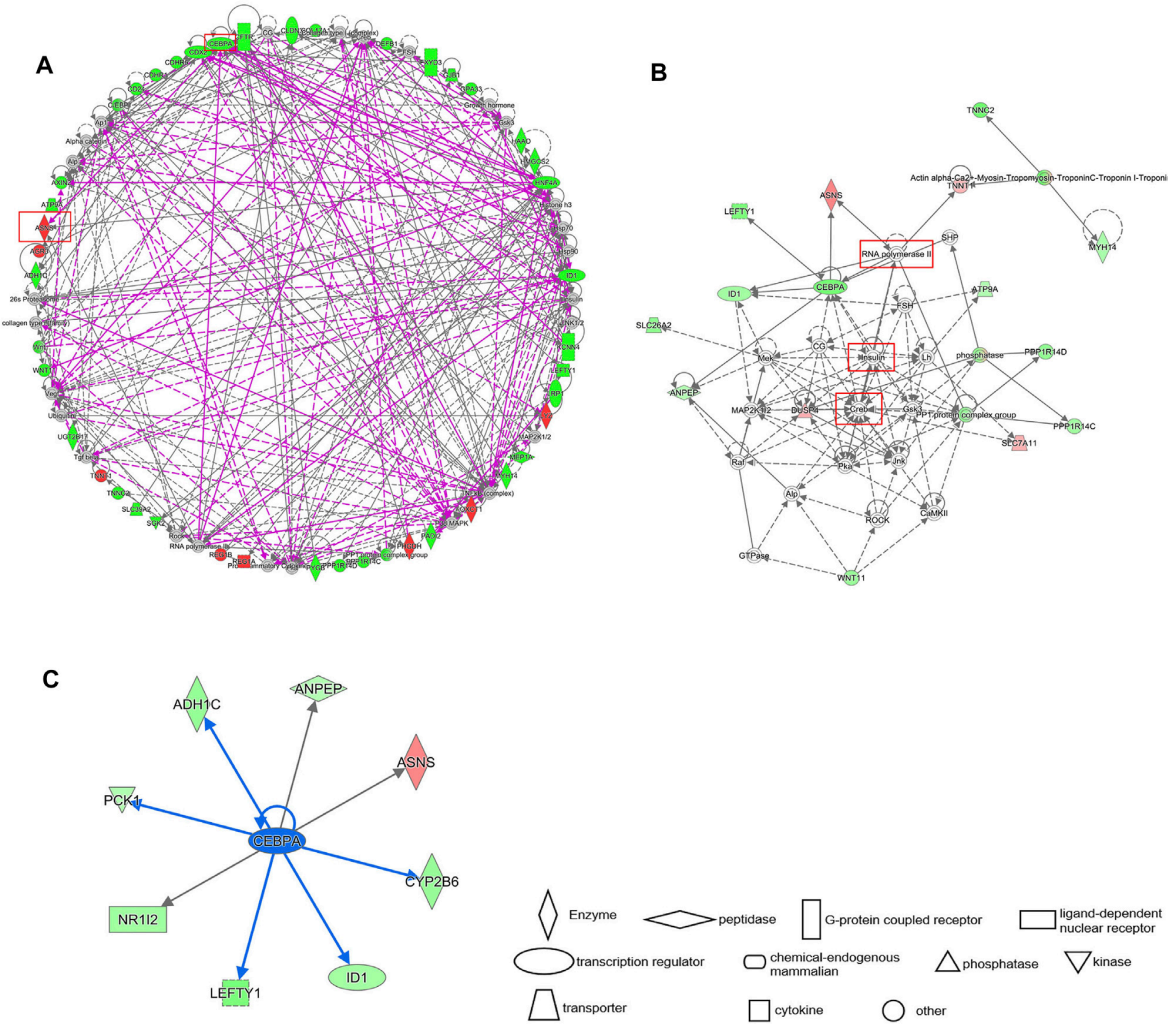
**(A)** IPA gene interaction network prediction diagram of differential genes. The prognostic genes ASNS and CEBPA are circled in red. Red represents up regulation and green represents down regulation. **(B)** The local interaction pathway diagram predicted by the IPA interaction network. The solid line represents the existing work verification, and the dash line represents the prediction. **(C)** The downstream genes of CEBPA. The blue arrow indicates inhibition.

The differential gene interaction network between Cluster 1 and Cluster 2 was constructed by IPA software. Based on IPA data of the identified molecular interactions reported in the literature, a gene pathway network containing ASNS and CEBPA emerged (Figure 8A). In our results, CREB, insulin, and RNA Pol II are critical nodes in the CEBPA- related pathway (Figure 8B). CREB, insulin, and RNA Pol II are the upstream of CEBPA, and their overexpression may down-regulate CEBPA. We further obtained all downstream differential genes of CEBPA, among which it is noteworthy that CEBPA, as an upstream gene of ASNS, upregulates the expression of ASNS (Figure 8C).

We clustered colon cancer samples in TCGA by the differential expression of ASNS, CEBPA, and CAD, and the results showed that the interaction between ASNS and CEBPA and their upstream and downstream genes was responsible for the differences in cell functional status and substance metabolism levels between Cluster 1 and Cluster 2.

### Preliminary Survey of the Difference in the Immune Microenvironment of the Two Clusters Stratified.

**FIGURE 9:**
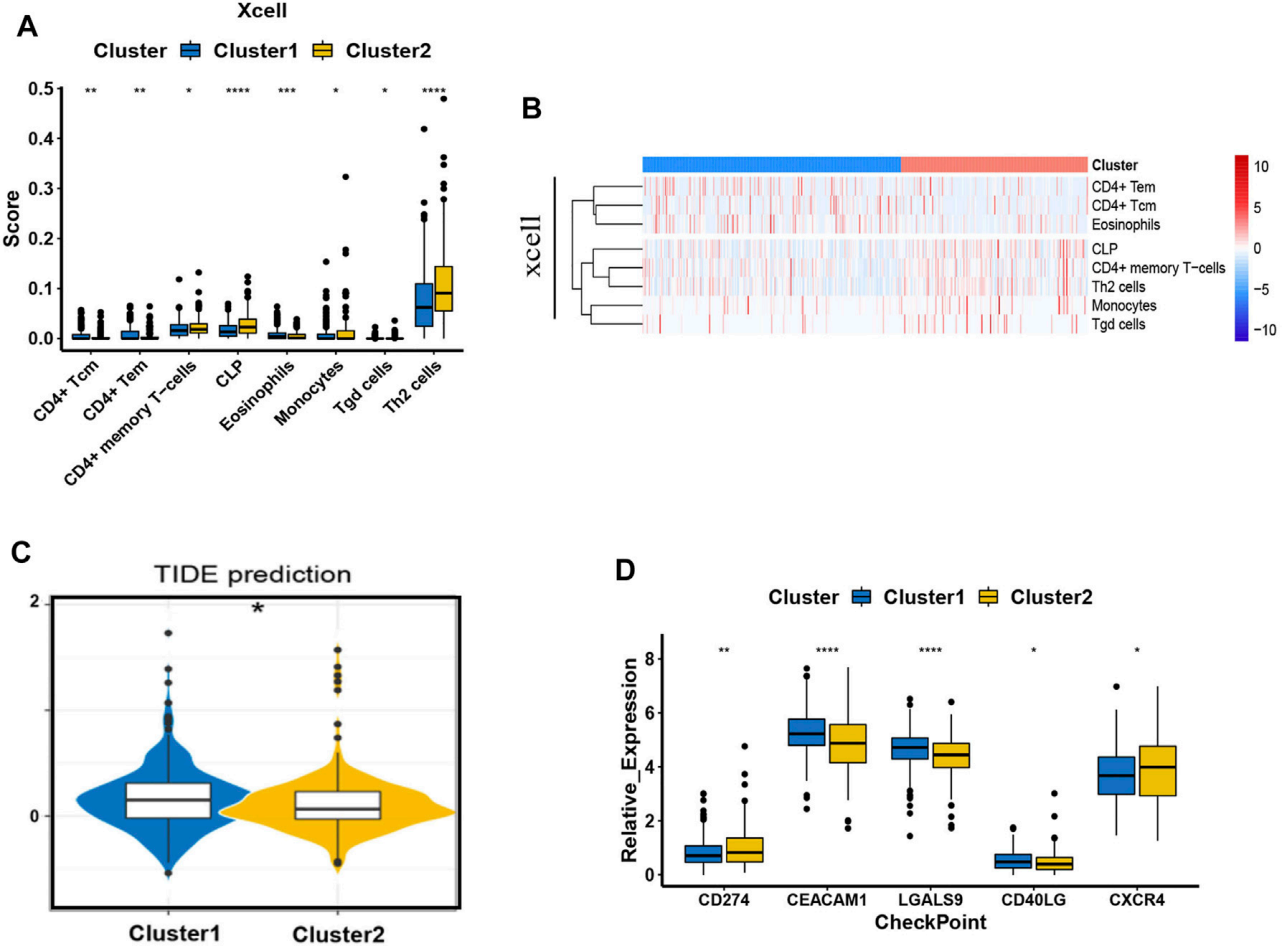
Immune behavior comparison between the two clusters. **(A)** The boxplots show the XCell scores for the enriched immunocytes. Within each group, the scattered dots represent values for cellular expression; whereas the thick line represents the median value. The statistical difference of the two clusters was compared using the Kruskal-Wallis test, and the asterisk above the box plot represents the degree of significance. **(B)** The heat map of the XCell immune infiltration analysis. **(C)** The boxplot representation of TIDE scores in Cluster 1 versus Cluster 2. **(D)** The expression pattern of five common types of immune checkpoints. ^*^ *p* < 0.05, ^**^ *p* < 0.01, ^***^ *p* < 0.001, and ^****^ *p* < 0.0001. Analyzed using unpaired *t*-test.

We have already confirmed that ASNS and CEBPA expression were associated with lymph node metastasis in colon cancer patients. To further assist the individual immunotherapy, we investigated the immune infiltrating and immune checkpoint genes in Cluster 1 and Cluster 2 samples. The differential immune infiltration features and the associated potential immunotherapeutic responses of the two clusters were explored by XCell and TIDE algorithm. The XCell software was employed to analyze the difference of the infiltrated immune cells between Cluster 1 and Cluster 2 in colon cancer samples from TCGA database. Th2 cells, CLP cells, and CD4^+^ memory T cells showed higher cell infiltration in Cluster 2; while CD4^+^ Tcm cells, CD4^+^ Tem cells, eosinophiles, monocytes, and Tgd cells were found to have the similar exhibition between the two clusters (Figures 9A,B). To link individual responses to immunotherapeutic responses, the TIDE algorithm was used. The lower TIDE score of Cluster 2 samples indicated that they may respond better to immunotherapy (Figure 9C). The expression levels of common immune checkpoints in Cluster 1 and Cluster 2 were also checked. CEACAM1, LGALS9, and CD40LG were highly expressed in Cluster 1, while CD274 (PD-L1) and CXCR4 were highly expressed in Cluster 2 (Figure 9D).

## Discussion

It has become evident that CRC is a highly heterogeneous disease with complex molecular pathogenesis. The classical TNM staging system benefits the clinical management of CRC for years. Consider the disappointing outcome of targeted drugs and immune checkpoint therapies on CRC patients; it is imperative to develop new biomarkers that enable the stratification of patients with CRC, which can guide the more-precise use of innovative therapies (Punt et al., 2017). Various central metabolic pathways, which intimately entwined with cell signaling and epigenetic networks, dysregulated in CRC cells. Moreover, the metabolic machineries and nutrient-sensing mechanisms orchestrated the behavior of immune cells located in the TME (Li et al., 2019).

In this study, three key genes, ASNS, CEBPA, and CAD, were screened by Cox regression analysis in colon cancer. ASNS consumes ATP to catalyze the conversion of aspartic acid and glutamine to asparagine and glutamate (Lomelino et al., 2017). Pyrimidines synthesis in cancerous proliferation can be regulated by enhancing aspartate availability for the enzymatic complex CAD. (Rabinovich et al., 2015). CEBPA, as a transcription factor, is a key regulator of many metabolic processes, such as lipid metabolism and amino acid metabolism (Diehl, 1998). Moreover, C/EBP homology protein negatively regulates the stress-dependent induction of the asparagine synthetase gene (Su and Kilberg, 2008).

TCGA data analysis and IHC staining of clinical tumor samples showed that ASNS and CEBPA tended to be underexpressed in the Cluster 2 with poor prognosis. Subsequently, we identified CEBPA as the upstream gene of ASNS, and showed a positive correlation between CEBPA and ASNS. To further reveal the underlying mechanism, we identified the upstream factors, i.e., CREB, Insulin, and RNA Pol II in the predicted signal network. CREB levels are generally positively correlated with tumor grade, stage, metastasis, increased recurrence rate, and poor prognosis (Steven et al., 2020). It has also been reported that CEBPA binds to CRE in the liver and antagonizes CREB (Roesler, 2000). And CREB often antagonizes insulin *in vivo* (Altarejos and Montminy, 2011). Insulin resistance is one of the factors that induce colon cancer and other tumors (McNabney and Henagan, 2017). Insulin can also activate CEBPA expression (Marques-Oliveira et al., 2018). Therefore, it can be speculated that CREB antagonizes the effect of insulin in colon cancer, reduces the activation and expression of CEBPA, and play a carcinogenic role. Furthermore, we found that CREB directly upregulated SLC7A11 expression, and CEBPA negatively correlated with SLC7A11. Studies have shown that SLC7A11 can promote cysteine absorption and glutathione biosynthesis, prevent oxidative stress, thus, significantly increase glutamine metabolism, and promote the growth of cancer cells (Koppula et al., 2018). It is possible that CREB up-regulation of SLC7A11 also play a carcinogenic role in colon cancer. Moreover, RNA Pol II was predicted to be an upstream factor associated with both CEBPA and ASNS. Rapid proliferation of tumor cells increases the expression of RNA Pol II. Transcription-coupled subpathway of nucleotide excision repair (NER) is activated by the stalling of RNA Pol II during transcriptional elongation (Marteijn et al., 2014). Our prediction model suggested that overexpression of RNA pol II down-regulated CEBPA and ASNS in Cluster 2.

The immune infiltration results showed that compared with the Cluster 1 patients, the degree of Th2 cell infiltration was much higher in Cluster 2 patients with a shorter survival time. In addition, the TIDE score of Cluster 2 patients was lower, indicating a better response to immunotherapy. It was also found that the expression of both CXCR4 and CD274 (PD-L1) in Cluster 2 were higher through the immune checkpoint analysis. Recent *PNAS* study reported that CXCR4 inhibition in combination with blockade of the PD-1/PD-L1 induced T cell infiltration and anticancer responses in human pancreatic and colorectal cancers (Biasci et al., 2020). Therefore, due to the high expression of CXCR4 and PD-L1 in Cluster 2, the synergistic treatment of anti-PD-1 and blocking CXCR4 targeting Cluster 2 patients may produce better therapeutic effects.

It was found that in our cluster model, ASNS and CEBPA play an important role in the prognosis of colon cancer patients, and patients with low ASNS and CEBPA expression had a worse prognosis, i.e., more prone to lymph node metastasis. Additionally, the expression profile of CAD was also associated with prognosis, i.e., patients with high CAD expression were more likely to metastasis and recurrence. CEBPA was the upstream gene of ASNS, and the expression of CEBPA and ASNS is positively correlated.We predict that upstream factors such as CREB, insulin, and RNA Pol II can regulate downstream genes, such as ASNS and SLC7A11, by affecting the expression of CEBPA, ultimately changing the cellular function and metabolism in colon cancer tissues. It even affects the infiltration of immune cells in TME, which may further affect the immunotherapy response of patients. Besides, these results suggested that aspartic acid metabolism play an important role in colon cancer and deserve further exploration.

## Conclusion

In summary, three genes associated with metabolic processes were selected as the key genes for risk clustering model establishment of colon cancer patients. The model was verified by molecular biological experiments performed on clinical specimens. The GO annotation and GSVA analysis were carried out to distinguish the pathway activities between the two clusters with OS differences. The signaling networks were predicted by IPA, and hub nodes were identified. These findings suggest that the key genes may serve as new prognostic markers for colon cancer. Moreover, the immune response comparison between the two clusters was also conducted to provide references for individualized immunotherapy.

## Data Availability

Publicly available datasets were analyzed in this study. This data can be found here: TCGA database: https://tcga-data.nci.nih.gov/tcga/, GEO database(GSE29623), https://www.ncbi.nlm.nih.gov/geo/.

## Abbreviations

ASNS, asparagine synthetase; BP, biological process; CAD, carbamoyl-phosphate synthetase 2, aspartate transcarbamylase, and dihydroorotase; CC, cellular components; CDF, cumulative distribution function; CEBPA, CCAAT/enhancer binding protein alpha; CLP, common lympoid progenitors; CRC, colorectal cancer; CRE, cAMP response element; CREB, cAMP response element-binding protein; CXCR4, CXC chemokine receptor 4; FDR, false discovery rate; GEO, gene expression omnibus; GO, gene ontology; GSVA, gene set variation analysis; HRP, horseradish peroxidase; IHC, immunohistochemistry; IPA, ingenuity pathway analysis; KEGG, Kyoto Encyclopedia of genes and genomes; NER, nucleotide excision repair; OS, overall survival; PD-1, programmed cell death protein 1; PD-L1, programmed death ligand 1; PPAR, peroxisome proliferators-activated receptors; RNA pol II, RNA polymerase II; ssGSEA, single sample gene set enrichment Analysis; TCGA, The cancer genome Atlas; Th2 cells, T-helper 2 cells; TIDE, tumor immune dysfunction and exclusion; TME, tumor microenvironment; TNM staging, Tumor-Node-Metastasis staging; t-SNE, t-distributed stochastic neighbor embedding; UC, urea cycle.

## References

[B1] Altarejos J. Y. Montminy M. (2011). CREB and The CRTC Co-Activators: Sensors For Hormonal and Metabolic Signals. Nat. Rev. Mol. Cel Biol 12 (3), 141–151. 10.1038/nrm3072 PMC4324555 21346730

[B2] Altman B. J. Stine Z. E. Dang C. V. (2016). From Krebs to Clinic: Glutamine Metabolism to Cancer Therapy. Nat. Rev. Cancer 16 (10), 619–634. 10.1038/nrc.2016.71 27492215 PMC5484415

[B3] Aran D. Hu Z. Butte A. J. (2017). xCell: Digitally Portraying the Tissue Cellular Heterogeneity Landscape. Genome Biol. 18 (1), 220. 10.1186/s13059-017-1349-1 29141660 PMC5688663

[B4] Biasci D. Smoragiewicz M. Connell C. M. Wang Z. Gao Y. Thaventhiran J. E. D. (2020). CXCR4 Inhibition in Human Pancreatic and Colorectal Cancers Induces an Integrated Immune Response. Proc. Natl. Acad. Sci. U S A. 117, 28960–28970. 10.1073/pnas.2013644117 33127761 PMC7682333

[B5] Diehl A. M. (1998). Roles of CCAAT/enhancer-Binding Proteins in Regulation of Liver Regenerative Growth. J. Biol. Chem. 273 (47), 30843–30846. 10.1074/jbc.273.47.30843 9812973

[B6] Freeman H. J. (2013). Early Stage Colon Cancer. World J. Gastroenterol. 19 (46), 8468–8473. 10.3748/wjg.v19.i46.8468 24379564 PMC3870492

[B7] Hänzelmann S. Castelo R. Guinney J. (2013). GSVA: Gene Set Variation Analysis for Microarray and RNA-Seq Data. BMC Bioinformatics 14, 7. 10.1186/1471-2105-14-7 23323831 PMC3618321

[B8] Jiang P. Gu S. Pan D. Fu J. Sahu A. Hu X. (2018). Signatures of T Cell Dysfunction and Exclusion Predict Cancer Immunotherapy Response. Nat. Med. 24 (10), 1550–1558. 10.1038/s41591-018-0136-1 30127393 PMC6487502

[B9] Keshet R. Szlosarek P. Carracedo A. Erez A. (2018). Rewiring Urea Cycle Metabolism in Cancer to Support Anabolism. Nat. Rev. Cancer 18 (10), 634–645. 10.1038/s41568-018-0054-z 30194362

[B10] Knott S. R. V. Wagenblast E. Khan S. Kim S. Y. Soto M. Wagner M. (2018). Asparagine Bioavailability Governs Metastasis in a Model of Breast Cancer. Nature 554 (7692), 378–381. 10.1038/nature25465 29414946 PMC5898613

[B11] Koppula P. Zhang Y. Zhuang L. Gan B. (2018). Amino Acid Transporter SLC7A11/xCT at the Crossroads of Regulating Redox Homeostasis and Nutrient Dependency of Cancer. Cancer Commun. 38 (1), 12. 10.1186/s40880-018-0288-x PMC5993148 29764521

[B12] Krebs H. A. (1970). The History of the Tricarboxylic Acid Cycle. Perspect. Biol. Med. 14 (1), 154–172. 10.1353/pbm.1970.0001 4923349

[B13] Lane A. N. Fan T. W.-M. (2015). Regulation of Mammalian Nucleotide Metabolism and Biosynthesis. Nucleic Acids Res. 43 (4), 2466–2485. 10.1093/nar/gkv047 25628363 PMC4344498

[B14] Li R. Qian J. Wang Y.-Y. Zhang J.-X. You Y.-P. (2014). Long Noncoding RNA Profiles Reveal Three Molecular Subtypes in Glioma. CNS Neurosci. Ther. 20 (4), 339–343. 10.1111/cns.12220 24393335 PMC6493123

[B15] Li X. Wenes M. Romero P. Huang S. C.-C. Fendt S.-M. Ho P.-C. (2019). Navigating Metabolic Pathways to Enhance Antitumour Immunity and Immunotherapy. Nat. Rev. Clin. Oncol. 16 (7), 425–441. 10.1038/s41571-019-0203-7 30914826

[B16] Lomelino C. L. Andring J. T. McKenna R. Kilberg M. S. (2017). Asparagine Synthetase: Function, Structure, and Role in Disease. J. Biol. Chem. 292 (49), 19952–19958. 10.1074/jbc.R117.819060 29084849 PMC5723983

[B17] Marques-Oliveira G. H. Silva T. M. Lima W. G. Valadares H. M. S. Chaves V. E. (2018). Insulin As A Hormone Regulator Of The Synthesis And Release Of Leptin By White Adipose Tissue. Peptides 106, 49–58. 10.1016/j.peptides.2018.06.007 29953915

[B18] Marteijn J. A. Lans H. Vermeulen W. Hoeijmakers J. H. J. (2014). Understanding Nucleotide Excision Repair and its Roles in Cancer and Ageing. Nat. Rev. Mol. Cel Biol 15 (7), 465–481. 10.1038/nrm3822 24954209

[B19] McNabney S. Henagan T. (2017). Short Chain Fatty Acids in the Colon and Peripheral Tissues: A Focus On Butyrate, Colon Cancer, Obesity and Insulin Resistance. Nutrients 9 (12), 1348. 10.3390/nu9121348 PMC5748798 29231905

[B20] Pascual G. Avgustinova A. Mejetta S. Martín M. Castellanos A. Attolini C. S.-O. (2017). Targeting Metastasis-Initiating Cells through the Fatty Acid Receptor CD36. Nature 541 (7635), 41–45. 10.1038/nature20791 27974793

[B21] Pavlova N. N. Hui S. Ghergurovich J. M. Fan J. Intlekofer A. M. White R. M. (2018). As Extracellular Glutamine Levels Decline, Asparagine Becomes an Essential Amino Acid. Cel Metab. 27 (2), 428–438. 10.1016/j.cmet.2017.12.006 PMC5803449 29337136

[B22] Punt C. J. A. Koopman M. Vermeulen L. (2017). From Tumour Heterogeneity to Advances in Precision Treatment of Colorectal Cancer. Nat. Rev. Clin. Oncol. 14 (4), 235–246. 10.1038/nrclinonc.2016.171 27922044

[B23] Rabinovich S. Adler L. Yizhak K. Sarver A. Silberman A. Agron S. (2015). Diversion of Aspartate in ASS1-Deficient Tumours Fosters De Novo Pyrimidine Synthesis. Nature 527 (7578), 379–383. 10.1038/nature15529 26560030 PMC4655447

[B24] Renner K. Singer K. Koehl G. E. Geissler E. K. Peter K. Siska P. J. (2017). Metabolic Hallmarks of Tumor and Immune Cells in the Tumor Microenvironment. Front. Immunol. 8, 248. 10.3389/fimmu.2017.00248 28337200 PMC5340776

[B25] Roesler W. J. (2000). What Is a cAMP Response Unit? Mol. Cel Endocrinol 162 (1-2), 1–7. 10.1016/s0303-7207(00)00198-2 10854692

[B26] Röhrig F. Schulze A. (2016). The Multifaceted Roles of Fatty Acid Synthesis in Cancer. Nat. Rev. Cancer 16 (11), 732–749. 10.1038/nrc.2016.89 27658529

[B27] Rubin A. L. (1990). Suppression of Transformation by and Growth Adaptation to Low Concentrations of Glutamine in NIH-3T3 Cells. Cancer Res. 50 (9), 2832–2839. 2328506

[B28] Simon K. (2016). Colorectal Cancer Development and Advances in Screening. Clin. Interv. Aging 11, 967–976. 10.2147/CIA.S109285 27486317 PMC4958365

[B29] Spinelli J. B. Yoon H. Ringel A. E. Jeanfavre S. Clish C. B. Haigis M. C. (2017). Metabolic Recycling of Ammonia via Glutamate Dehydrogenase Supports Breast Cancer Biomass. Science 358 (6365), 941–946. 10.1126/science.aam9305 29025995 PMC5748897

[B30] Steven A. Friedrich M. Jank P. Heimer N. Budczies J. Denkert C. (2020). What Turns CREB on? And off? And Why Does it Matter? Cell. Mol. Life Sci. 77 (20), 4049–4067. 10.1007/s00018-020-03525-8 32347317 PMC7532970

[B31] Su N. Kilberg M. S. (2008). C/EBP Homology Protein (CHOP) Interacts with Activating Transcription Factor 4 (ATF4) and Negatively Regulates the Stress-dependent Induction of the Asparagine Synthetase Gene. J. Biol. Chem. 283 (50), 35106–35117. 10.1074/jbc.M806874200 18940792 PMC2596379

[B32] Vander Heiden M. G. Cantley L. C. Thompson C. B. (2009). Understanding the Warburg Effect: The Metabolic Requirements of Cell Proliferation. Science 324 (5930), 1029–1033. 10.1126/science.1160809 19460998 PMC2849637

[B33] Ward P. S. Thompson C. B. (2012). Metabolic Reprogramming: A Cancer Hallmark Even Warburg Did Not Anticipate. Cancer Cell 21 (3), 297–308. 10.1016/j.ccr.2012.02.014 22439925 PMC3311998

[B34] Wilkinson N. W. Yothers G. Lopa S. Costantino J. P. Petrelli N. J. Wolmark N. (2010). Long-Term Survival Results of Surgery Alone Versus Surgery Plus 5-Fluorouracil and Leucovorin For Stage II and Stage III Colon Cancer: Pooled Analysis of NSABP C-01 through C-05. A Baseline from Which to Compare Modern Adjuvant Trials. Ann. Surg. Oncol. 17 (4), 959–966. 10.1245/s10434-009-0881-y 20082144 PMC2935319

[B35] Zhang J. Fan J. Venneti S. Cross J. R. Takagi T. Bhinder B. (2014). Asparagine Plays a Critical Role in Regulating Cellular Adaptation to Glutamine Depletion. Mol. Cel 56 (2), 205–218. 10.1016/j.molcel.2014.08.018 PMC4224619 25242145

