# Supplementary figures and images for: Identification of Key Genes Related With Aspartic Acid Metabolism and Corresponding Protein Expression in Human Colon Cancer With Postoperative Prognosis and the Underlying Molecular Pathways Prediction
Source: Front Cell Dev Biol. 2022 Jan 31;10:812271. doi: 10.3389/fcell.2022.812271 (PMC8841526; doi:10.3389/fcell.2022.812271)

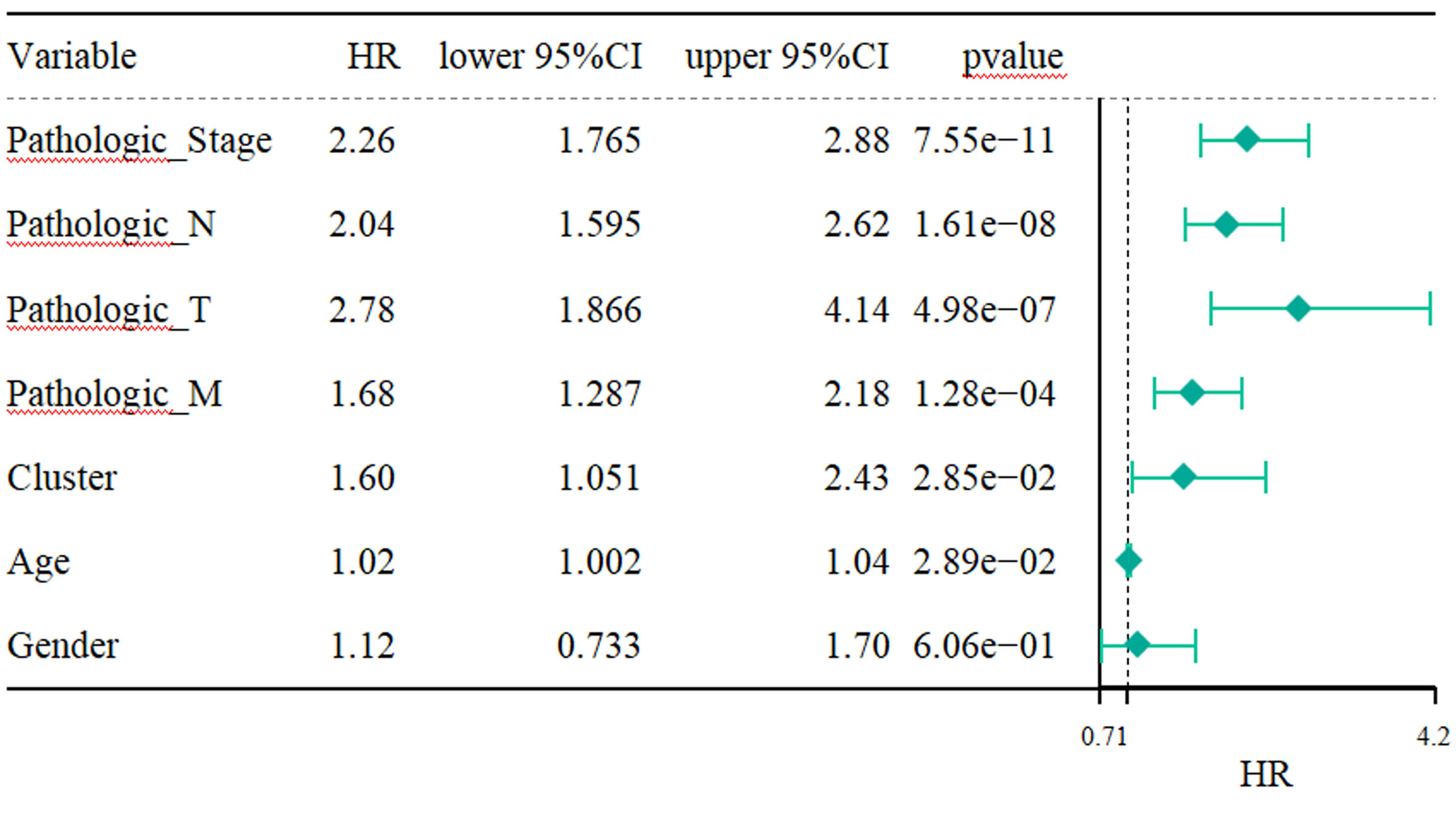

Supplement: Supplementary file 1 [file Image3.JPEG]

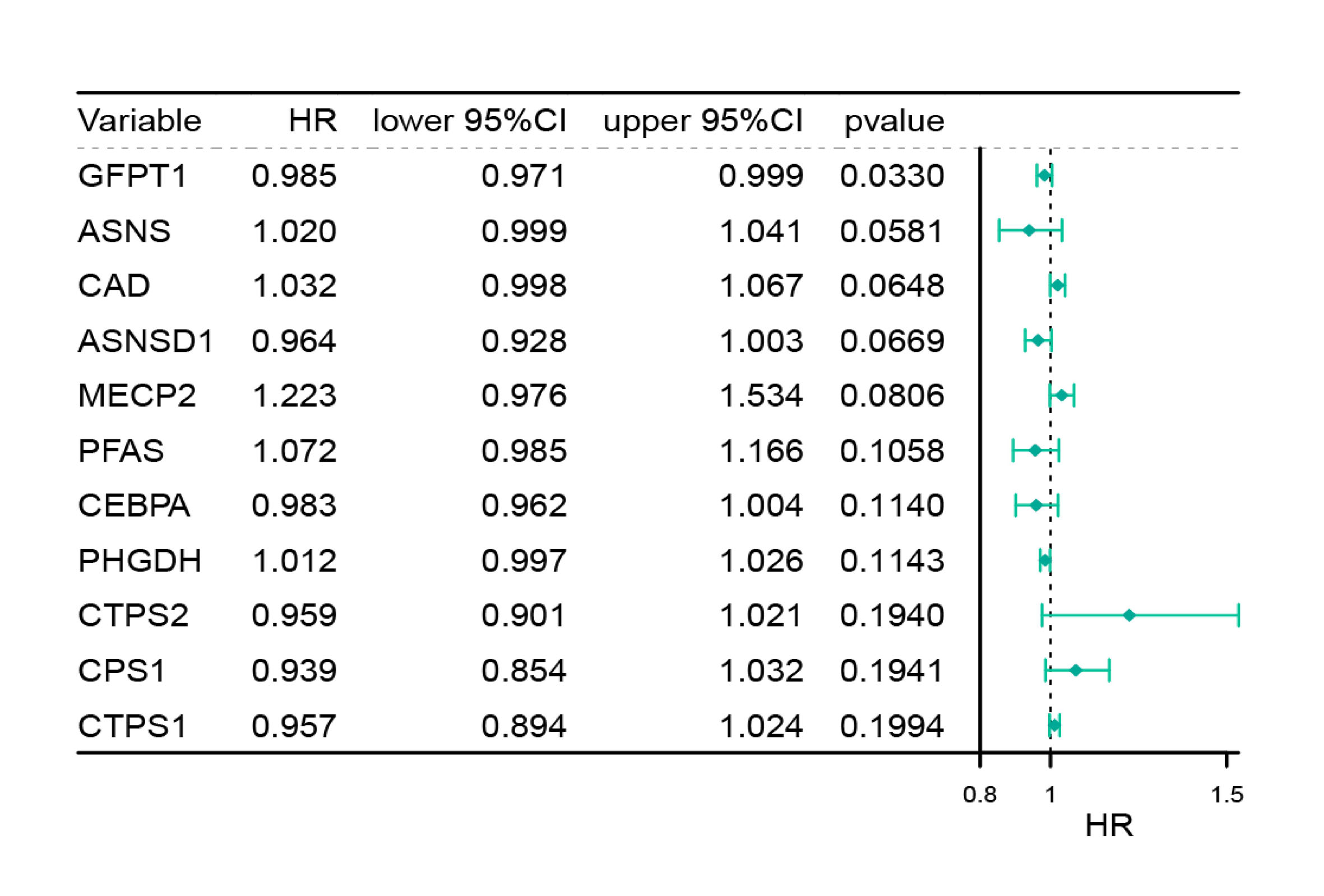

Supplement: Supplementary file 2 [file Image1.JPEG]

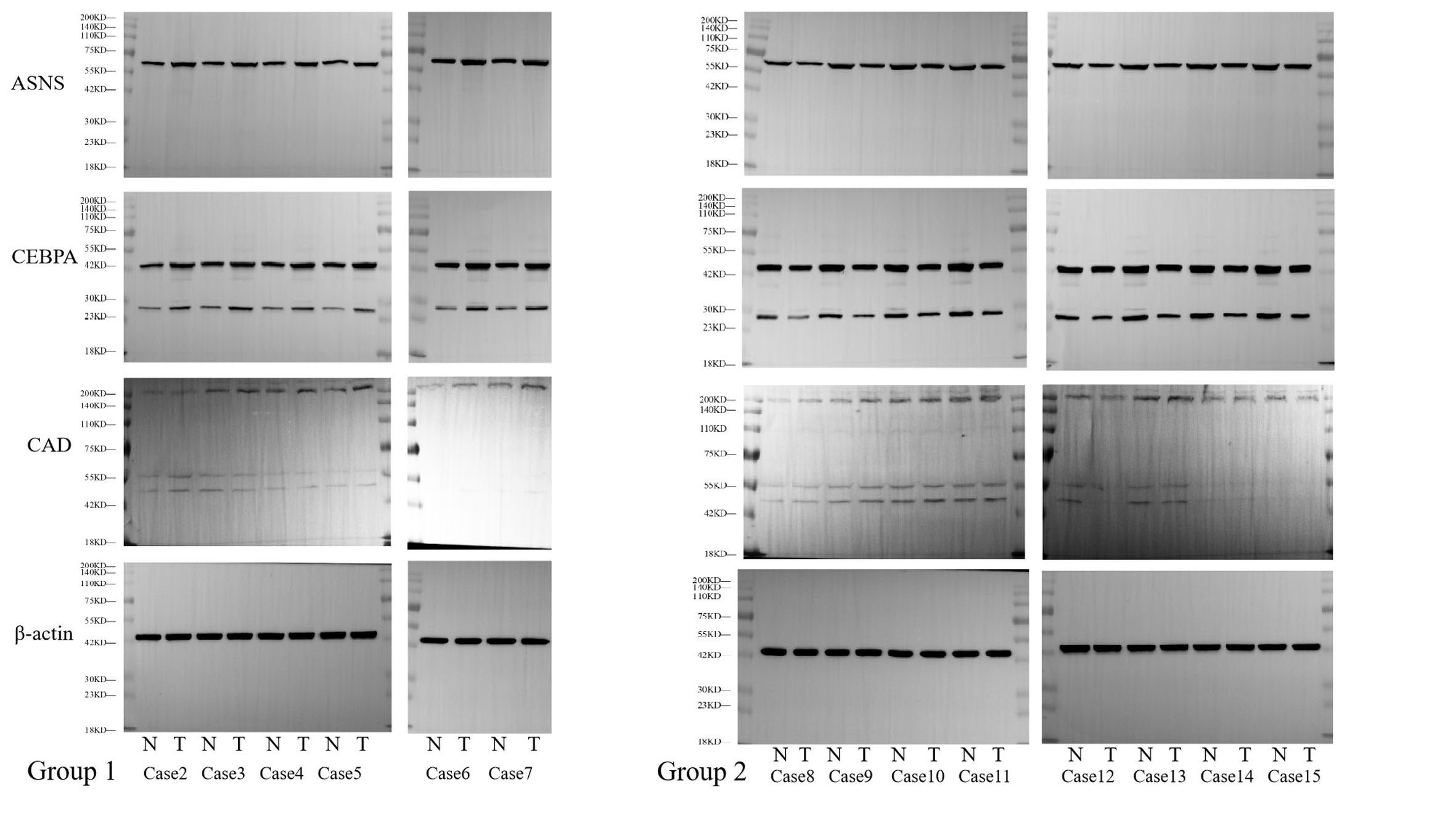

Supplement: Supplementary file 3 [file Image4.JPEG]

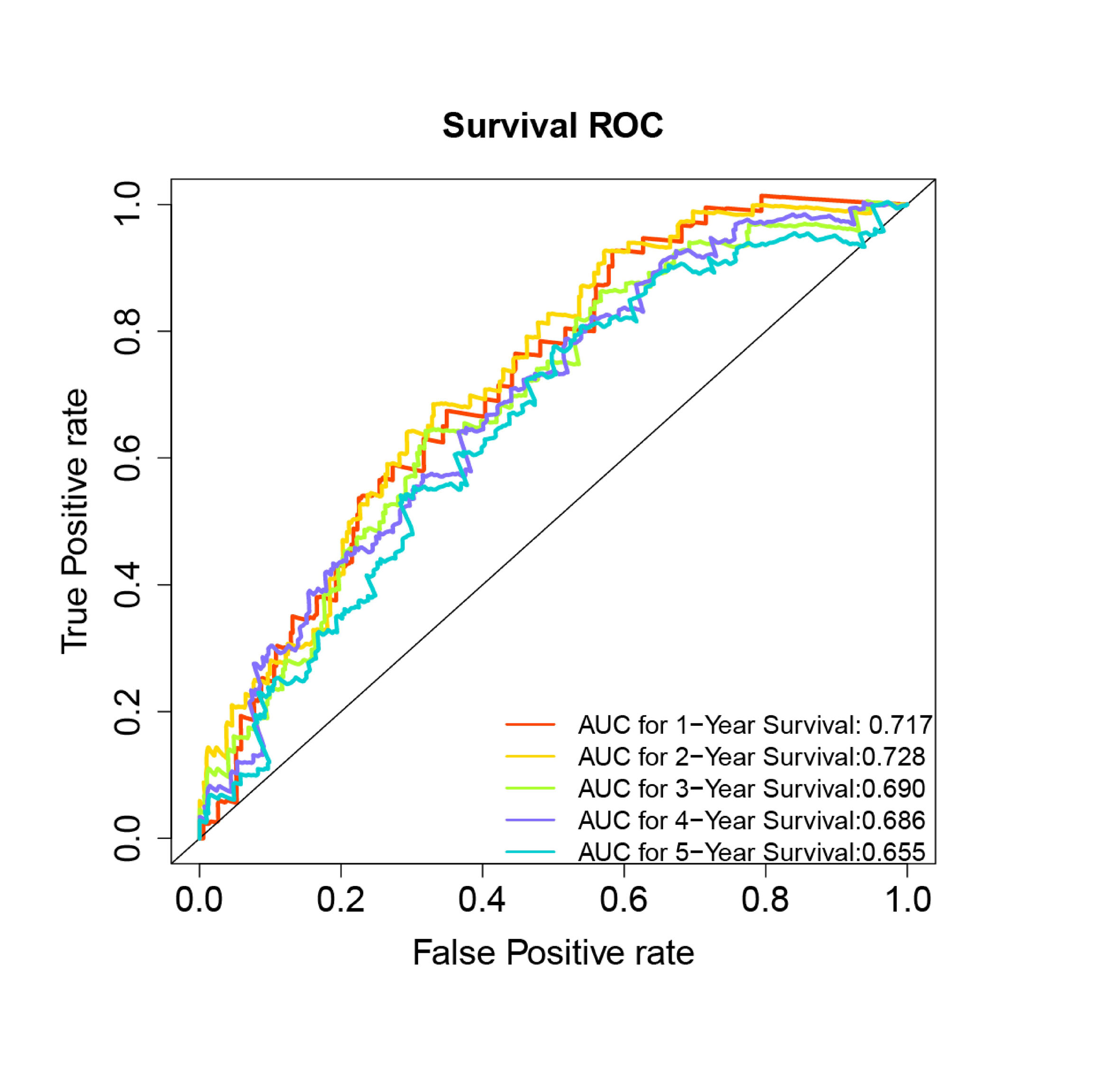

Supplement: Supplementary file 4 [file Image2.JPEG]
